# White-matter abnormalities in presymptomatic *GRN* and *C9orf72* mutation carriers

**DOI:** 10.1093/braincomms/fcac333

**Published:** 2022-12-19

**Authors:** Hyunwoo Lee, Ian R A Mackenzie, Mirza Faisal Beg, Karteek Popuri, Rosa Rademakers, Dana Wittenberg, Ging-Yuek Robin Hsiung

**Affiliations:** Division of Neurology, Department of Medicine, University of British Columbia, Vancouver V6T2B5, Canada; Department of Pathology and Laboratory Medicine, Faculty of Medicine, University of British Columbia, Vancouver V6T2B5, Canada; School of Engineering Science, Simon Fraser University, Burnaby V5A1S6, Canada; Department of Computer Science, Memorial University of Newfoundland, St John’s A1B3X5, Canada; Applied and Translational Neurogenomics, VIB Center for Molecular Neurology, VIB, Antwerp 2610, Belgium; Department of Biomedical Sciences, University of Antwerp, Antwerp 2610, Belgium; Department of Neuroscience, Mayo Clinic, Jacksonville, FL 32224, USA; Division of Neurology, Department of Medicine, University of British Columbia, Vancouver V6T2B5, Canada; Division of Neurology, Department of Medicine, University of British Columbia, Vancouver V6T2B5, Canada

**Keywords:** frontotemporal dementia, MRI, white matter

## Abstract

A large proportion of familial frontotemporal dementia is caused by TAR DNA-binding protein 43 (transactive response DNA-binding protein 43 kDa) proteinopathies. Accordingly, carriers of autosomal dominant mutations in the genes associated with TAR DNA-binding protein 43 aggregation, such as Chromosome 9 open reading frame 72 (*C9orf72*) or progranulin (*GRN*), are at risk of later developing frontotemporal dementia. Brain imaging abnormalities that develop before dementia onset in mutation carriers may serve as proxies for the presymptomatic stages of familial frontotemporal dementia due to a genetic cause. Our study objective was to investigate brain MRI-based white-matter changes in predementia participants carrying mutations in *C9orf72* or *GRN* genes. We analysed mutation carriers and their family member controls (noncarriers) from the University of British Columbia familial frontotemporal dementia study. First, a total of 42 participants (8 *GRN* carriers; 11 *C9orf72* carriers; 23 noncarriers) had longitudinal T_1_-weighted MRI over ∼2 years. White-matter signal hypointensities were segmented and volumes were calculated for each participant. General linear models were applied to compare the baseline burden and the annualized rate of accumulation of signal abnormalities among mutation carriers and noncarriers. Second, a total of 60 participants (9 *GRN* carriers; 17 *C9orf72* carriers; 34 noncarriers) had cross-sectional diffusion tensor MRI available. For each participant, we calculated the average fractional anisotropy and mean, radial and axial diffusivity parameter values within the normal-appearing white-matter tissues. General linear models were applied to compare whether mutation carriers and noncarriers had different trends in diffusion tensor imaging parameter values as they neared the expected age of onset. Baseline volumes of white-matter signal abnormalities were not significantly different among mutation carriers and noncarriers. Longitudinally, *GRN* carriers had significantly higher annualized rates of accumulation (estimated mean: 15.87%/year) compared with *C9orf72* carriers (3.69%/year) or noncarriers (2.64%/year). A significant relationship between diffusion tensor imaging parameter values and increasing expected age of onset was found in the periventricular normal-appearing white-matter region. Specifically, *GRN* carriers had a tendency of a faster increase of mean and radial diffusivity values and *C9orf72* carriers had a tendency of a faster decline of fractional anisotropy values as they reached closer to the expected age of dementia onset. These findings suggest that white-matter changes may represent early markers of familial frontotemporal dementia due to genetic causes. However, *GRN* and *C9orf72* mutation carriers may have different mechanisms leading to tissue abnormalities.

## Teaser

Lee *et al*. report that MRI-based white-matter abnormalities are more pronounced in presymptomatic *GRN* and *C9orf72* mutation carriers, compared with family controls who do not carry the mutations. They suggest that white-matter changes may represent early markers of familial frontotemporal dementia associated with a genetic cause.

## Graphical Abstract

**Graphical abstract:**
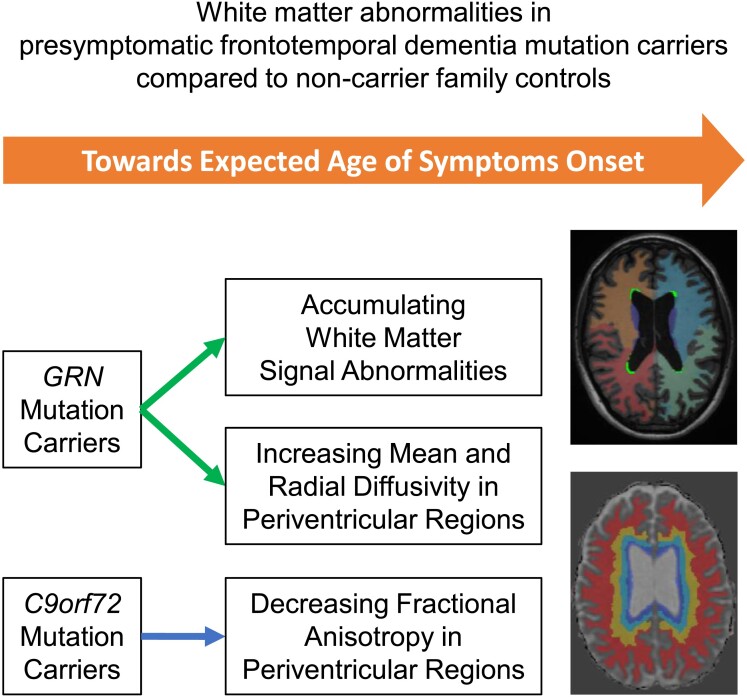

## Introduction

Frontotemporal dementia is a common cause of presenile dementia, clinically presented with a heterogeneous range of behavioural and personality changes (behavioural variant frontotemporal dementia) or speech and language difficulties (primary progressive aphasia).^1^ Around 30% of all frontotemporal dementia cases are known to be familial, often caused by tauopathies or TAR DNA-binding protein 43 (TDP-43) proteinopathies.^2^ Therefore, individuals who carry autosomal dominant mutations in the genes associated with tauopathies [e.g. carriers of mutations in microtubule-associated protein tau (*MAPT+*)] or TDP-43 proteinopathies [e.g. progranulin (*GRN+*) or chromosome 9 open reading frame 72 (*C9orf72+*)] have a predictable risk for developing frontotemporal dementia in the future. Accordingly, abnormal brain imaging changes observed in relatively young and asymptomatic mutation carriers may be proxies for the presymptomatic stages of familial frontotemporal dementia due to a genetic cause.

MRI studies have demonstrated that structural imaging abnormalities are apparent decades prior to expected symptom onset, with changes in the white-matter (WM) preceding those in the grey matter (GM).^2^ WM signal abnormalities (WMSAs) are a common MRI marker of WM tissue status, which denotes the region of altered fat/water ratio due to a variety of causes, including small vessel disease or an inflammatory environment. WMSAs are typically measured in terms of hyperintensities (T_2_-HyperWMSA) on fluid-attenuated inversion recovery (FLAIR) or T_2_-weighted MRI, although similar regions may also appear as hypointensities (T_1_-HypoWMSA) on gradient-echo T_1_-weighted MRI.^3^ T_2_-HyperWMSAs are prominent in symptomatic *GRN*+ carriers compared with *C9orf72*+ or *MAPT*+ carriers, largely affecting the frontal and occipital regions as well as parts of the temporal and parietal lobes.^4–7^ Therefore, it may be hypothesized that in *GRN*+ carriers, the development of WMSAs begins at some point during the presymptomatic stage and progresses throughout the disease course at a rate higher than that in other mutation subtypes or in normal aging. This was investigated in a previous multi-centre study (The Genetic Frontotemporal Dementia Initiative, GENFI), but the authors did not find evidence of higher T_2_-HyperWMSA accrual rate in presymptomatic *GRN*+ carriers relative to those who do not carry the specific mutation (‘noncarriers’).^7^ Furthermore, diffusion tensor imaging (DTI), which provides measures of WM microstructural integrity based on the magnitude and direction of tissue water diffusion,^8^ have shown different patterns of presymptomatic WM integrity decline across the mutation carrier subgroups.^9–18^ The locations of the altered fibres generally coincide with those affected in symptomatic carriers,^19,20^ suggesting that the deviation from the normal WM structure begins at the presymptomatic phase of familial frontotemporal dementia.

In this study, we assessed the accumulation of WMSAs and the alterations of DTI parameters in predementia carriers of *GRN* and *C9orf72* mutations, leveraging MRI data from the University of British Columbia (UBC) familial frontotemporal dementia study.^21^ We hypothesized that (i) *GRN*+ carriers would have a higher rate of WMSA accrual compared with *C9orf72*+ carriers, or family members who do not carry the mutations (*C9orf72*− and *GRN*−; noncarriers) and that (ii) the level of DTI parameter alterations would be higher in *GRN*+ and *C9orf72*+ carriers compared with the noncarriers. It is recognized that WMSAs have significantly altered DTI parameters [e.g. lower fractional anisotropy (FA) and/or higher mean diffusivity (MD)] compared with normal-appearing WM tissues (NAWM; i.e. WM areas not affected by signal abnormalities) and that they can affect the interpretation of DTI findings if not adjusted for.^22,23^ Therefore, we also specifically assessed whether the DTI parameters within the NAWMs become altered in the mutation carriers versus the noncarriers.

## Materials and methods

### Participants

Participants were recruited from the UBC familial frontotemporal dementia study, which is an ongoing single-centre longitudinal observational study of the Canadian cohort of familial frontotemporal dementia associated with TDP-43 pathology. Study inclusion and exclusion criteria were described previously.^21^ The study was approved by the UBC Clinical Ethics Review Board. All participants provided written, informed consent. Genetic testing and the recruitment protocol for this cohort have been described previously.^21,24,25^ Study recruitment began in January 2006 and a total of 131 participants were enrolled as of February 2022. Of those, a total of 112 participants had at least 1.5T MRIs done as of February 2022. However, because of staggered entry into the study, not every participant had completed the full study protocol at the time of this analysis. Accordingly, we analysed two subsets of the participants selected based on the availability of appropriate data: (i) for the longitudinal accumulation of WMSA, *N* = 42 participants with both baseline and 2 years of longitudinal follow-up T_1_-weighted MRIs [two scans per participant; average interval of 28 months ± standard deviation (SD) = 8] and (ii) for the cross-sectional analysis of DTI-based WM abnormalities, *N* = 60 participants with DTI scans (one scan per participant). For this analysis, we only included the participants who were not classified into the dementia category, as determined by a consensus meeting involving the study neurologist and neuropsychologist.

### Genetic status

Study participants were screened for mutations related to familial frontotemporal dementia and then stratified into mutation carriers (*C9orf72*+ or *GRN*+) or noncarriers (*C9orf72*− or *GRN*−) based on DNA extracted from peripheral blood, according to the previously described protocols.^26,27^ Every *C9orf72*+ participant had expansions that were at least 100 repeats in length. Throughout the study, the participants and researchers remained blind to the genetic mutation status.

### MRI methods

#### MRI acquisition

All MRI data were acquired on a 1.5T GE Signa scanner at the UBC Hospital MRI Research Centre, using the following imaging parameters: (i) localizers (0:25 min): sagittal/coronal and axial; fast gradient repetition time (TR): 5.4, echo time (TE): 1.6, one average, the field of view (FOV): 22 cm, 256 × 128 matrices; (ii) 3D T_1_-fast spoiled gradient-echo IR prepped (8:35 min): TR/TE (ms) = 10.3/4.8, 8° flip angle, 166 × 256 × 256, 1.0 × 0.98 × 0.98 mm^3^, FOV: 166; (iii) proton density/T_2_ dual (4:00 min), axial, TR = 2800, TE = 30/90, 90° flip angle, 256 × 256 × 35, 0.86 × 0.86 × 5.0 mm^3^, FOV 220; (iv) DTI (11:42 min): axial, spin-echo echo-planar imaging, TR = 13 000 ms TE = 85 ms, asset: 21 000 db, tensor: 25 diff directions, two averages, freq directions (DIR): R/L, phase encoding (PE) DIR: posterior-anterior (PA), number of excitations (NEX) = 2, 256 × 256 × 48, 1.25 × 1.25 × 2.50 mm^3^, FOV: 320.

#### MRI processing

T_1_-weighted images were visually checked for quality and then processed using the FreeSurfer 5.3 pipeline, which provides cortical reconstruction and volumetric segmentation.^28,29^ We utilized a version implemented in the ‘Cloud Engine Resource for Accelerated Medical Image Computing for Clinical Applications’ portal (CERAMICCA; https://ceramicca.ensc.sfu.ca). All FreeSurfer outputs were manually examined and corrected for errors. Total intracranial volumes (TIVs) for subsequent statistical analyses were calculated using a multi-atlas label fusion technique, described in detail elsewhere.^30^

While WMSAs are conventionally represented by T_2_-HyperWMSAs, the limited resolution of our T_2_-weighted images (with a slice thickness of 5 mm) rendered them less suitable for the segmentation of hyperintense WM voxels. As an alternative indicator of WMSA, we employed T_1_-HypoWMSAs that were segmented as part of the FreeSurfer volumetric outputs. Previously, a study using a similar MRI platform (3D-fast spoiled gradient-echo IR prepped sequence on a 1.5T GE scanner) has shown that the volumes of FreeSurfer-derived WM hypointensities were strongly correlated with the volumes of hyperintensities on FLAIR images.^31^ In that context, we considered the T_1_-HypoWMSAs as a nonspecific marker of abnormal WM tissues.

DTI images were processed using the FMRIB Software Library Diffusion Toolbox.^32,33^ Briefly, the steps included: (i) manual checking of the diffusion data, (ii) brain extraction on the nondiffusion weighted b0 images using ‘bet’, (iii) eddy currents and subject motion correction using ‘eddy’ and (iv) voxel-wise fitting of the eddy-corrected diffusion tensor data using ‘dtifit’. These steps produced the maps of FA (the degree of anisotropic diffusion within WM fibre tracts) and MD (the magnitude of water diffusion within tissue), as well as radial diffusivity (RaD; a measure of myelin change) and axial diffusivity (AxD; a measure of axonal damage) maps for each participant.

**Figure 1:**
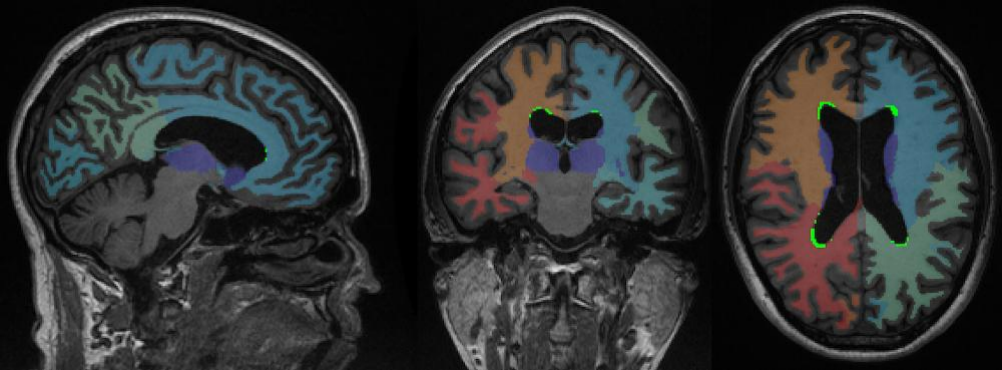
**Example of the WM lobar region of interests, with T_1_-HypoWMSAs overlaid in green.** Colours indicate different lobar segmentations.

**Figure 2:**
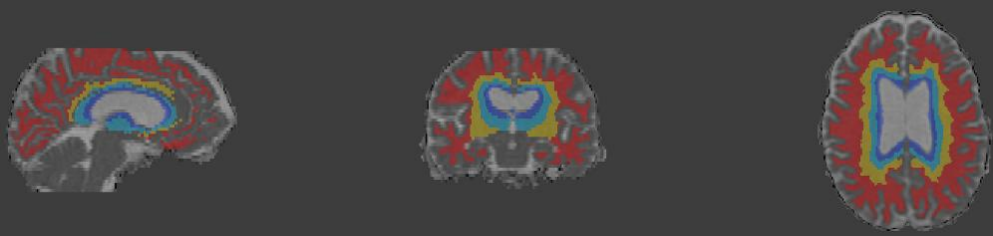
**Example of the WM radial layer region-of-interests, with a MD image overlaid.** In radially outward order, Blue: periventricular layer; Light blue and Yellow: medial layer; Red: peripheral layer.

Regional variations in WM tissue abnormalities were assessed in terms of predefined region-of-interest (ROI) comparisons. Specifically, we subdivided the FreeSurfer-generated cerebral WM volumes into lobes and radial layers, similar to the approach used in previous studies.^6,7^ The lobes (frontal, temporal, parietal and occipital) were defined by combining the corresponding Desikan–Killiany atlas labels through FreeSurfer’s ‘mri_annotation2label’ and ‘mri_aparc2aseg’ functions.^34^  Figure 1 provides an example of the lobar ROIs. The radial layers were obtained by determining the normalized distance between the ventricular edge and the cortex-WM boundary and then dividing the WM into four equidistant layers. As in Sudre *et al*.,^7^ we combined the two middle layers to obtain a total of three layers (i.e. Layer 1: periventricular; Layers 2 + 3: medial; Layer 4: peripheral). Figure 2 provides an example of the radial layers.

### Statistical analysis

#### Participants characteristics

All analyses were conducted using SAS PROC GLM and JMP software (SAS, Cary, NC, USA). The one-way ANOVA was used to compare the mean age, education years, mean age of symptoms onset in family and expected years to symptom onset (EYO; a proxy term for how close a participant is to developing frontotemporal dementia symptoms,^35^ calculated by subtracting the participant’s age at scan from the mean age of symptom onset in their respective family) among *GRN*+, *C9orf72*+ and noncarriers. Differences in the sex ratio were assessed using the likelihood-ratio *χ*^2^ test. The mini-mental state examination (MMSE) and the frontal assessment battery (FAB) test scores were compared using the Kruskal–Wallis test as they were not normally distributed.

#### Baseline differences in T_1_-HypoWMSA burden (cross sectional)

A general linear model (GLM) was used to compare the baseline burden of whole-brain WMSA among *GRN*+, *C9orf72*+ and noncarriers. For each participant, we calculated the baseline burden of T_1_-HypoWMSAs as the fraction of the TIV [WMSA fraction (WMSAf)] to account for the differences in head sizes using the ‘proportion’ method.^36^ The calculated whole-brain WMSA fraction (WMSAf) were used as the dependent variable in the following GLM,

(1) WMSAf∼CarrierSubgroup+Age+Sex+EYO

The CarrierSubgroup term included *GRN*+, *C9orf72*+ and noncarriers (pooled *GRN*− and *C9orf72*−). Age is a significant risk factor for cerebral small vessel disease,^37^ which appears as WMSAs on MRI. EYO was included to examine whether the rate of WMSA accumulation would be higher in participants getting closer to the family mean age of onset.

Additionally, we calculated the WMSAf within each lobe (bilateral frontal, temporal, parietal and occipital) to explore the regional distribution of T_1_-HypoWMSAs. We used a subject-specific random-effects model to compare the baseline WMSA burden among the lobes.

#### Differences in the annualized rates of the total T_1_-HypoWMSA accumulation (longitudinal)

For each participant, the longitudinal rates of the total T_1_-HypoWMSA accumulation were expressed in terms of the annualized percentage change relative to the baseline volume. The annualization factor was obtained by dividing 12 months by the months between the baseline and the follow-up MRI visits,

(2) $$\frac{\mathrm{WMSAvo}l_{\mathrm{Baseline}}-\mathrm{WMSAvo}l_{\mathrm{Followup}\_2y}}{\mathrm{WMSAvo}l_{\mathrm{Baseline}}}\times 100\times \mathrm{Annualization}\;\mathrm{factor}$$

The following GLM was used to compare the annualized percentage change rates of the whole-brain T_1_-HypoWMSA accumulation among *GRN*+, *C9orf72*+ and noncarriers,

(3) %ChangeinWMSA∼CarrierSubgroup+Age+Sex+EYO+TIV+WMSAf

Definitions of the CarrierSubgroup, age, sex and EYO terms were identical to the abovementioned cross-sectional WMSA model. For this model, we have adjusted for the impact of head size variability by including TIVs as a covariate (i.e. ‘GLM’ approach).^36^ Additionally, we have included the baseline burden of WMSAs as they were expected to be predictive of the longitudinal WMSA progression; we have used WMSAf as the covariate as it led to reduced multicollinearity among the covariates as well as more normally distributed GLM model residuals.

For both WMSA models, we first conducted an omnibus *F*-test to examine whether the model outperformed the null model. After confirming the significance of the model, we assessed an effect test of the CarrierSubgroup term followed by *post hoc* pairwise tests (three total: *GRN*+ versus noncarriers, *C9orf72*+ versus noncarriers and *GRN*+ versus *C9orf72*+) using the Tukey’s HSD method to account for multiple comparisons. *P* < 0.05 was considered statistically significant.

#### Differences in the alterations of NAWM DTI parameters (cross sectional)

For each participant, we calculated the average FA, MD, RaD and AxD parameter values within the periventricular, medial and peripheral layers. Specifically, we focused on the NAWM regions to investigate the differences in WM microstructure that are not captured by the T_1_-HypoWMSAs. This was done by masking out the T_1_-HypoWMSA regions during the calculation of DTI parameters.

The following GLM was fitted for the FA and MD values within each radial layer,

(4) DTIParameter∼CarrierSubgroup+EYO+CarrierSubgroup×EYO+Age+Sex+WMVf+WMSAf

The definitions of the CarrierSubgroup, EYO, age and sex terms were identical to the WMSA GLM models. The CarrierSubgroup × EYO interaction term was included to assess whether the mutation carriers had different ‘slopes’ of DTI parameter change over the EYO range. WMSA fractions at the time of the DTI scan were also included, as it is possible that the NAWM penumbra regions surrounding the focal T_1_-HypoWMSA have altered DTI parameters due to more widespread WM tissue injury.^38,39^ WM volume fraction (WMVf; a measure of WM atrophy) was calculated by dividing the total cerebral WM volume by the TIV; this was included as WM atrophy was expected to influence the DTI parameters.^40^

Like the WMSA models, we first examined whether the omnibus *F*-test was significant and then tested whether the slopes were significantly different between the *GRN*+ or *C9orf72*+ carriers versus the noncarriers. Additionally, to investigate the rates of diffusion along the longitudinal and perpendicular axes, we conducted similar GLM-based group comparisons of the RaD and AxD parameters in the WM layers that exhibited significant group differences in the FA or MD parameters.

## Results

### Participant characteristics

The participants’ characteristics are outlined in Table 1 (those who were included in the WMSA analysis) and Table 2 (those who were included in the DTI analysis). Years of education, mean age of onset in families, MMSE and FAB scores were not significantly different among the subgroups. Although ANOVA did not reveal significant subgroup differences in age and EYO, the *C9orf72*+ carriers in our cohort tended to be younger and therefore further away from the expected symptoms onset. The sex ratio was imbalanced, with more female *GRN*+ carriers in the study and also more female *C9orf72+* carriers who contributed to the DTI analysis.

**Table 1:** Participant characteristics for those included in the WMSA analysis: demographics, neuropsychological scores and family information

Analysis: WMSA	Participants included in the WMSA analysis		
Mutation subgroup	*GRN*+	*C9orf72*+	Noncarriers (*GRN*− and *C9orf72*−)
# of included participants	8	11	23
Baseline age in years, mean ± SD	49.5 ± 9.1	46.7 ± 9.4	52.2 ± 8.0
Sex ratio, M:F	1:7	6:5	11:12
Years of education, mean ± SD	12.3 ± 1.4	14.2 ± 3.5	13.4 ± 2.9
MMSE at baseline, mean ± SD	29.3 ± 1.0	29.5 ± 0.8	29.3 ± 0.9
FAB at baseline, mean ± SD	16.8 ± 1.0	16.7 ± 1.9	17.3 ± 1.1
Mean age of onset in family in years, mean ± SD	57.9 ± 3.2	56.8 ± 6.4	57.8 ± 6.1
EYO, median, [IQR], (range)	−6.5 [−18 to −1.25] (−23 to 4)	−6 [−17 to −2] (−37 to −1)	−6 [−8 to −2] (−28 to 14)

Age, MMSE and FAB indicate the average values at baseline. IQR = interquartile range.

**Table 2:** Participant characteristics for those included in the DTI analysis: demographics, neuropsychological scores and family information

Analysis: DTI	Participants included in the DTI analysis		
Mutation subgroup	*GRN*+	*C9orf72*+	noncarriers (*GRN*− and *C9orf72*−)
# of included participants	9	17	34
Baseline age in years, mean ± SD	53.8 ± 6.8	47.5 ± 9.4	53.6 ± 10.2
Sex ratio, M:F	1:8	6:11	18:16
Years of education, mean ± SD	13.0 ± 3.0	14.2 ± 3.2	13.2 ± 2.7
MMSE at baseline, mean ± SD	29.0 ± 1.0	29.4 ± 0.9	29.1 ± 1.0
FAB at baseline, mean ± SD	17.2 ± 1.2	16.9 ± 1.6	16.6 ± 2.0
Mean age of onset in family in years, mean ± SD	58.4 ± 3.4	58.8 ± 7.7	58.3 ± 5.4
EYO, median, [IQR], (range)	−5 [−8.3 to 1] (−18 to 6)	−5 [−21.5 to −1.5] (−37 to 4)	−3.5 [−7.25 to 1.25] (−33 to 14)

Overlapping participants: 7 *GRN*+, 11 *C9orf72*+ and 21 noncarriers contributed to both WMSA and DTI analyses. Age, MMSE and FAB indicate the average values at the time of DTI. IQR = interquartile range.

### Baseline differences in T_1_-HypoWMSA burden (cross sectional)

Although not statistically significant, it should be noted that our *GRN*+ group had overall smaller T_1_-HypoWMSA volumes at baseline, likely due to the female predominance in the group. Adjusting for age, sex and EYO, baseline WMSAfs were not significantly different among the subgroups after correcting for multiple comparisons using Tukey’s HSD method (model-adjusted group comparisons are reported in Table 3).

**Table 3:** Comparison of T_1_-HypoWMSA burden at baseline, corrected for age, sex and estimated years to onset

Region	Mutation subgroup	Baseline T_1_-HypoWMSA volume in fraction of TIV, marginal mean ± SE
Whole brain	GRN+	0.12 ± 0.01
	*C9orf72*+	0.13 ± 0.01
	Noncarriers	0.12 ± 0.008

T1-HypoWMSA burden was calculated as the fraction of the TIV. *GRN*+, *C9orf72*+ and noncarriers were not significantly different. SE = standard error.

**Figure 3:**
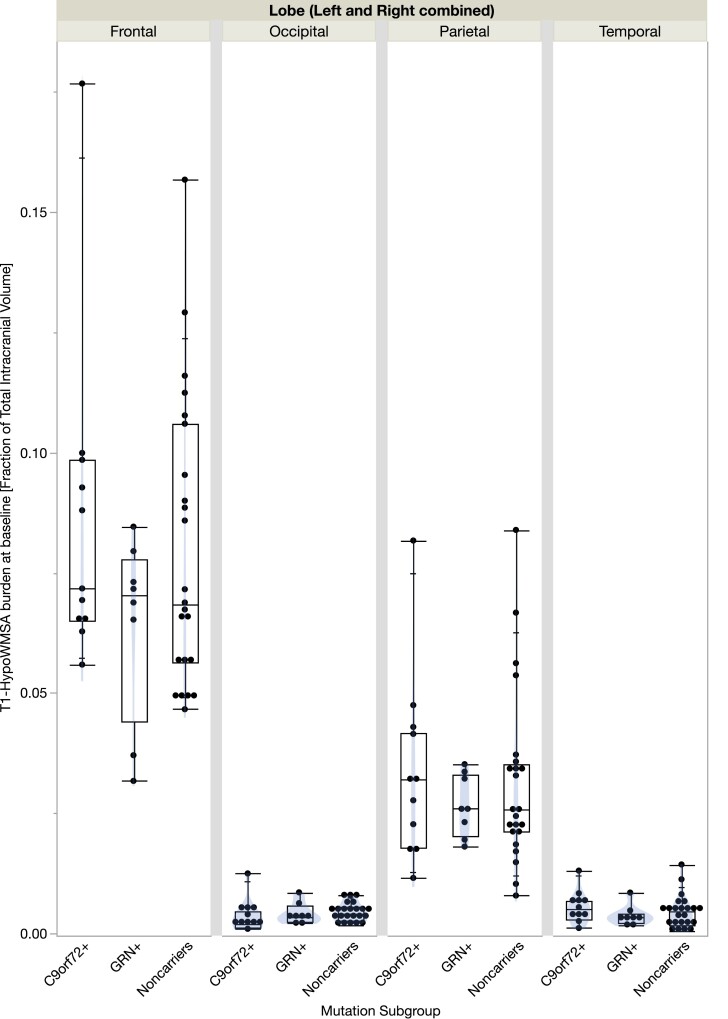
**WM lobar-wise distribution of the T_1_-HypoWMSA burden at baseline, calculated as the fraction of the TIV.** Boxplots indicate quantiles. Individual dots indicate individual participant data. A subject-specific random-effects model was used to compare the baseline WMSA burden among the lobes. The frontal lobe had significantly higher T_1_-HypoWMSA burden compared with the parietal, temporal, or occipital lobes (*P* < 0.0001). The parietal lobe had a significantly higher T_1_-HypoWMSA burden compared with the temporal or occipital lobes (*P* < 0.0001). Baseline burden was not significantly different between the temporal and occipital lobes.

WM lobar-wise analysis suggested that the frontal lobe had significantly higher T_1_-HypoWMSA burden compared with the parietal, temporal or occipital lobes (*P* < 0.0001) across the subgroups. Also, the parietal lobe had a significantly higher T_1_-HypoWMSA burden compared with the temporal or occipital lobes (*P* < 0.0001). The baseline burden was not significantly different between the temporal and occipital lobes. Figure 3 summarizes the findings.

### Differences in the annualized rates of the total T_1_-HypoWMSA accumulation (longitudinal)

We found significant differences in the annualized rates of T_1_-HypoWMSA accumulation among the subgroups; the mean estimated rates were highest in *GRN*+ (15.87%/year), followed by *C9orf72*+ (3.69%/year) and noncarriers (2.64%/year). *Post hoc* pairwise comparisons indicated that the rates of WMSA accumulation in *GRN*+ were significantly higher compared with those in *C9orf72*+ (*P* = 0.0038) or noncarriers (*P* = 0.02), with ‘very large’ (Cohen’s *d* > 1.4) effect sizes.^41^ The rates were not significantly different between *C9orf72*+ and noncarriers. Overall, the rates were not significantly associated with age, sex, EYO and TIV terms. As expected, a lower WMSAf at baseline was associated with higher percentage changes in T_1_-HypoWMSAs. The estimated rates are summarized in Table 4.

**Table 4:** Whole-brain T_1_-HypoWMSA progression: estimated annualized rates of total cerebral T_1_-HypoWMSA accumulation by mutation subgroup, adjusted for age, sex, EYO, TIV and baseline WMSAf

Region	Mutation subgroup	Estimated annualized % change from the baseline, marginal mean ± SE, [95% CI]	*Post hoc* Tukey’s HSD, mean difference, [95% CI], (Cohen’s *D*), [95% CI]
Whole brain	*GRN+*	15.87 ± 3.25 [9.26, 22.48]	*GRN* + versus noncarriers *P* = 0.0038* 13.22 [3.93, 22.53] (1.59) [0.61, 2.54] *GRN*+ versus *C9orf72*+ *P* = 0.020* 12.18 [1.67, 22.68] (1.46) [0.38, 2.51] *C9orf72*+ versus noncarriers *P* = 0.94 1.05 [−6.93, 9.04] (0.13) [−0.64, 0.89]
	*C9orf72*+	3.69 ± 2.65 [−1.69, 9.08]	
	Noncarriers	2.64 ± 1.82 [−1.06, 6.34]	

*Post hoc* group-wise comparisons were conducted using the Tukey’s HSD method and significant differences are marked by an asterisk. CI, confidence interval; SE, standard error.

### Differences in the alterations of NAWM DTI parameters (cross sectional)

We observed significant group effects in the comparison of FA and MD parameters in the periventricular NAWM layer, but not in the medial and peripheral layers. In particular, the effect was driven by the interaction between the mutation subgroup term and the EYO term, suggesting that increasing EYO differentially affected periventricular FA and MD indices among mutation carriers and noncarriers. We also noted a significant group-by-EYO interaction term in the periventricular RaD parameter, but not in the AxD parameter. These findings are summarized in Table 5.

**Table 5:** Periventricular DTI parameters at baseline: group-wise comparison of the [subgroup: EYO] interaction terms estimated from GLM tested whether the association between periventricular DTI indices and increasing EYO differed between mutation carriers and noncarriers

DTI index	Differences in the interaction ‘slopes’ between carriers and noncarriers, estimated mean ± SE, [95% CI]
FA (periventricular)	*GRN* + versus noncarriers *P* = 0.39 −0.00063 ± 0.00073 [−0.0021, 0.00084] *C9orf72+*versus noncarriers *P* = 0.0040* −0.0011 ± 0.00038 [−0.0019, −0.00038]
Mean Diffusivity (Periventricular)	*GRN*+ versus noncarriers *P* = 0.02* 6.06-E06 ± 2.44E-06 [1.15E-06, 1.10E-05] *C9orf72 +* versus noncarriers *P* = 0.26 1.44E-06 ± 1.26E-06 [−1.10E-06, 3.97E-06]
Radial Diffusivity (Periventricular)	*GRN*+ versus noncarriers *P* = 0.02* 5.69E-06 ± 2.30E-06 [1.06E-06, 1.03E-05] *C9orf72 +* versus noncarriers *P* = 0.09 2.08E-06 ± 1.19E-06 [−3.13E-07, 4.47E-06]

Significant differences are marked by an asterisk.

Periventricular FA: We found a significant interaction between the CarrierSubgroup and the EYO terms (*P* = 0.004). Specifically, the interaction ‘slopes’ were significantly more negative in *C9orf72*+ compared with noncarriers, suggesting a faster decline of FA values with increasing EYO. This was not observed between *GRN*+ and noncarriers. Age, sex and baseline WMSAf terms were not associated with FA values. A smaller WMVf was associated with a lower FA value.

Periventricular MD: We found a significant interaction between the CarrierSubgroup and the EYO terms (*P* = 0.02). Specifically, the interaction ‘slopes’ were significantly more positive in *GRN*+ compared with noncarriers, suggesting a faster increase of MD values with increasing EYO. This was not observed between *C9orf72*+ and noncarriers. Sex and baseline WMSAf terms were not associated with MD values while increasing age was significantly associated with increasing MD values. A smaller WMVf was associated with a higher MD value.

Periventricular RaD: A significant interaction was found between the CarrierSubgroup and the EYO terms (*P* = 0.02), where the interaction ‘slopes’ were significantly more positive in *GRN*+ compared with noncarriers. This suggested a faster increase of RaD values with increasing EYO in *GRN*+ compared with noncarriers, but not in *C9orf72*+. Sex and baseline WMSAf terms were not associated with RaD values, but increasing age was significantly associated with increasing RaD values. A smaller WMVf was associated with a higher RaD value.

## Discussion

In this study, we compared carriers of *GRN* or *C9orf72* mutations and their non-carrier family members in terms of the longitudinal accumulation of T_1_-HypoWMSAs and the cross-sectional alterations of DTI parameters. Baseline burden of T_1_-HypoWMSAs was not significantly different among *GRN*+, *C9orf72*+ and noncarriers. Longitudinally, the annualized rates of T_1_-HypoWMSA accumulation were significantly higher in *GRN*+ compared with *C9orf72*+ and noncarriers, indicating more rapid progression of visible WM abnormalities in *GRN*+. Also, DTI analysis revealed that *GRN*+ carriers with a higher EYO (i.e. closer to expected symptom onset) were associated with higher MD and RaD values. On the other hand, *C9orf72*+ carriers with higher EYO were associated with lower FA values. These findings suggest that WM abnormalities progress differently in *GRN*+ and *C9orf72*+ carriers during the predementia stages of pathogenesis.

To explore the spatial distribution of WM abnormalities at baseline, we first cross sectionally compared the volumes of T_1_-HypoWMSAs within the WM lobar regions. For all subgroups, the vast majority of the T_1_-HypoWMSAs were found in the frontal and parietal lobes, while distributions within the occipital and temporal lobes were relatively less. Overall, there were no significant differences in the baseline volumes of T_1_-HypoWMSAs among the subgroups, which is in line with findings from the GENFI study, where the authors reported nonsignificant differences in T_2_-HyperWMSA volumes among presymptomatic *GRN*+, *C9orf72*+, *MAPT*+ and noncarriers.^6,7^ However, as the burden of WMSAs is more extensive in symptomatic *GRN*+ carriers,^4–7^ it can be expected that the average predementia rate of WMSA progression would be higher in *GRN*+.

As expected, our *GRN*+ carriers had significantly higher rates of total T_1_-HypoWMSA accumulation compared with *C9orf72*+ or noncarriers. Interestingly, our longitudinal finding was unlike that from the GENFI study, where the authors reported nonsignificant differences in the rates of T_2_-HyperWMSA accumulation between presymptomatic *GRN*+ and noncarriers.^7^ We propose potential reasons for the discrepancy. First, the two studies utilized different WMSA measures: T_1_-HypoWMSAs versus T_2_-HyperWMSAs. Even though these two measures tend to be strongly correlated, T_2_-weighted sequences are relatively more sensitive to WM alterations and can detect higher volumes of WMSAs.^31^ Yet, T_1_-HypoWMSAs may represent more focused areas of severe WM injury in which progression is associated with cognitive decline.^42^ Second, our *GRN*+ cohort was potentially closer to symptom onset compared with the GENFI cohort, as implied by the median EYO of −6.5 years and a higher average baseline burden of WMSAs [1342.9 mm^3^ (T_1_-Hypo) versus 925.9 mm^3^ (T_2_-Hyper); the difference would be even greater if the sequences are matched]. Third, our study utilized a single 1.5T MRI scanner, whereas the GENFI study utilized 3T MRI scanners. As 3T scanners are generally more sensitive to WMSAs,^43,44^ the GENFI study protocol likely had the ability to detect more WMSA volumes compared with our protocol. For these reasons, it is difficult to directly compare our findings to the GENFI findings, although both studies demonstrate the vulnerability of WM in predementia *GRN*+ carriers.

The causes of WMSAs in our predementia *GRN*+ carriers remain to be elucidated due to the unavailability of pathological correlates. Nevertheless, previous histopathological studies have shown significant microglial activation and dystrophy in frontotemporal dementia patients with *GRN* mutation, with a higher proportion of the amoeboid shape within more severely abnormal areas.^45–47^ In particular, more severely affected T_2_-HyperWMSA areas exhibited a greater degree of myelin loss and astrogliosis with minimal infarcts or haemorrhages,^47^ suggesting that the WMSAs may be inflammatory mediated rather than secondary to vascular events. Whether this explanation also applies to our *GRN*+ carriers requires further verification, although anecdotally, our cohort was relatively young with an average age of around 50 years. Also, we found that the vast majority of the T_1_-HypoWMSA accumulation occurred within the periventricular layer, which, compared with the deep WM regions, may be more susceptible to inflammatory responses following blood–brain barrier disruption.^48^

A higher burden of WMSAs is associated with GM volume loss in symptomatic *GRN*+ carriers,^7,47^ suggesting a potential relationship between WM abnormalities and tissue atrophy. Yet, the temporal relationship between WMSAs and brain atrophy in frontotemporal dementia remains less understood. Cortical and subcortical GM volume loss is relatively more pronounced in *C9orf72*+ during predementia stages,^25^ although changes in *GRN*+ are also noted in the frontal and the parietal lobes.^49,50^ But once in the symptomatic stage, *GRN*+ carriers suffer the fastest rates of brain volume loss, led by accelerated decline in the frontal, temporal and parietal lobe.^50,51^ A possible hypothesis is that the rapidly accumulated WMSAs during the predementia stages lead to subsequent secondary degenerations (e.g. Wallerian or retrograde degeneration) of the affiliated tissues, which may contribute to the future accelerated rates of lobar atrophy in *GRN*+ carriers. For example, Wallerian degeneration in the central nervous system progresses for months to years,^52^ suggesting that the consequences of WMSA accumulations during predementia on atrophy may be delayed and not manifested immediately. Furthermore, an asymmetric pattern of GM atrophy, which variably affects the left or right hemisphere, has been reported in symptomatic *GRN*+ carriers as well as asymptomatic *GRN*+ carriers nearing symptomatic onset.^35,51,53^ A previous GENFI study noted that in symptomatic mutation carriers, asymmetry in the frontal lobar GM atrophy is associated with the asymmetric distribution of frontal lobar T_2_-HyperWMSA; however, the authors did not find any association in asymptomatic mutation carriers.^7^ This suggests that an uneven lateral distribution of WM abnormalities may partly explain asymmetric GM atrophy, although their sequential relationship remains unclear. A longitudinal analysis of the individuals who convert from asymptomatic to symptomatic frontotemporal disorders is warranted to answer whether an early asymmetric progression of WMSA leads to future brain atrophy preferentially affecting the same hemisphere. Another point to consider is the conceivable impact of the increased inflammatory responses (e.g. build-up of extracellular water and/or increase in the number of inflammatory cells) on the brain volume of *GRN*+ carriers. If significant, this effect may confound the interpretation of brain volume changes upon the initiation of treatments that resolve the inflammatory environment, in terms of ‘pseudoatrophy’, which refers to transient brain volume loss not due to tissue atrophy, but due to resolution of oedema and gliosis.^54^ Monitoring of brain tissue water change, for example using water-sensitive MRI sequences, may help elucidate this possibility.

In addition to the visibly abnormal tissues, we explored microstructural properties within the NAWM regions using conventional DTI parameters. The tested GLM showed significant effects within the periventricular WM layer (where most of the WMSA accumulation occurred), specifically a relationship between increasing EYO (i.e. getting closer to the expected symptom onset age) and increasing MD and RaD in *GRN*+ but not in *C9orf72*+ carriers. This indirectly reflects the progressive increase in free diffusion over the predementia disease course, potentially related to the exacerbation of inflammatory-mediated gliosis and demyelination described in *GRN*+.^47^ Furthermore, the peri-WMSA NAWM may represent the ‘penumbra’ that is perturbed by the severely abnormal foci,^38^ and predisposed to conversion to WMSAs in the future.^40,55^ Longitudinal monitoring of the DTI parameters would be required to answer this question.

Intriguingly, there was no evidence of decreasing FA with increasing EYO in *GRN*+ compared with noncarriers, which is similar to a previous longitudinal finding.^56^ As FA is more influenced by axonal health than by myelin,^57^ our finding suggests relative sparing of axons within the NAWM, despite presumed demyelination, over the predementia disease course. This is in line with the pathological description of mild axonal loss in *GRN*+^47^ and may explain the absence of volume reduction at baseline in our *GRN*+ carriers.^25^

In contrast, our *C9orf72*+ carriers were characterized by a relationship between increasing EYO and decreasing FA. At the same time, there was no evidence of increasing MD over the predementia course, which, along with the longitudinal stability of T_1_-HypoWMSAs, suggests that WM abnormalities in our *C9orf72*+ carriers are less likely to be due to cellularity and oedema-related changes but more likely to be due to declining axonal density and/or membrane integrity. Indeed, the baseline analysis of our *C9orf72*+ arriers found cortical and subcortical volume reductions compared with *GRN*+,^25^ potentially reflecting atrophy associated with compromised axons.

Our study had several limitations that laid the foundation for future work. First, being a single-centre study, our sample size was unbalanced, especially with a smaller number of *GRN*+ carriers with a female preponderance. In that sense, our findings involving the *GRN*+ group may have been confounded by sex-related differences on WMSA and DTI measures. Second, our longitudinal analysis was based on two time points, restricting us to the use of a linear model. Although our scan interval was relatively short, WM abnormalities over the long term may progress in a non-linear pattern, particularly approaching the time of symptoms onset.^35^ A longer follow-up study, including symptomatic converters, is necessary to determine whether the courses of WMSAs differ between lobes as they approach the time of symptom onset. Similarly, our DTI analysis was cross sectional, allowing us to capture only a snapshot of the predementia course and assess the progression of DTI indices only indirectly in terms of EYO, not actual time. Additional follow-up of our cohort will alleviate these two issues in the future. Third, a direct comparison of our T_1_-HypoWMSA and more conventionally used T_2_-HyperWMSA measures must be done carefully. While hypointensities on T_1_-weighted 3D gradient-echo images generally correspond to hyperintensities on T_2_-weighted images, it likely detects relatively smaller volumes of WMSAs (e.g. average 10% difference in non-demented elderly).^31^ In that sense, our definition of NAWM may have encompassed the areas that may have been classified as hyperintensities on T_2_-weighted images. Although T_1_-HypoWMSAs and T_2_-HyperWMSAs tend to be highly correlated, their exact relationships remain less understood. In particular, it needs to be verified in the future whether the two measures follow similar or different trajectories in mutation carriers approaching symptomatic conversion. Fourth, our diffusion images had a relatively lower resolution, which is prone to partial volume effects. It is possible that our DTI findings, especially those from the periventricular regions, may have been influenced by cerebrospinal fluid contamination of brain voxels. Moreover, our findings based on predefined ROIs may have been relatively less sensitive to WM abnormalities due to averaging the signal over multiple regions. Future analyses are warranted to investigate more specific locations of the WM abnormalities. Fifth, our conventional DTI parameters need to be interpreted with caution, as the results could have been confounded by the presence of extracellular water. This may be addressed in the future by using post-processing techniques, such as free-water imaging,^58^ that can yield separate signals from ‘free-water’ (associated with freely diffusing water in the extracellular compartment) and ‘tissue compartment’ (associated with tissue-constrained water, i.e. free-water corrected DTI indices).

Despite these limitations, our study indicates the ongoing presence of WM alterations in predementia *GRN* and *C9orf72* mutation carriers. Notably, our *GRN*+ carriers had significantly higher rates of T_1_-HypoWMSA accumulation as well as an increasing tendency of MD and RaD along with increasing EYO. These may reflect the initial phases of the inflammatory environment and the vulnerability to demyelination and gliosis described in frontotemporal dementia patients with the *GRN* mutation. Even though there was no evidence of WMSA or diffusivity changes in our *C9orf72*+ carriers, a decreasing tendency of FA along with increasing EYO may reflect axonal vulnerability, potentially associated with a higher degree of volume loss observed in these carriers compared with *GRN*. Accordingly, our findings suggest that WM changes could represent early neuroimaging markers of familial frontotemporal dementia due to a genetic cause and that different mutation carriers may require different targeted preventative measures or treatments.

## Data Availability

De-identified clinical, imaging and molecular data will be made available at the Canadian Association of Research Library (http://www.carl-abrc.ca/).

## Abbreviations

AxD =: axial diffusivity
C9orf72 =: chromosome 9 open reading frame 72
DTI =: diffusion tensor imaging
EYO =: expected years to symptom onset
FA =: fractional anisotropy
FAB =: frontal assessment battery
FLAIR =: fluid-attenuated inversion recovery
FOV =: field of view
GENFI =: the genetic frontotemporal dementia initiative
GLM =: general linear model
GM =: grey matter
GRN =: progranulin
MAPT =: microtubule-associated protein tau
MD =: mean diffusivity
MMSE =: mini-mental state examination
NAWM =: normal-appearing white matter
RaD =: radial diffusivity
ROI =: region of interest
TDP-43 =: TAR DNA-binding protein 43
TE =: echo time
TIV =: total intracranial volume
TR =: repetition time
Tukey’s HSD test =: Tukey’s honest significance test
T_1_-HypoWMSA =: white-matter hypointensities on gradient-echo T_1_-weighted MRI
T_2_-HyperWMSA =: white-matter hyperintensities on fluid-attenuated inversion recovery or T_2_-weighted MRI
WM =: white matter
WMSA =: white-matter signal abnormality
WMSAf =: white-matter signal abnormality fraction
WMVf =: white-matter volume fraction
